# IL-33 promotes MHC class II expression in murine mast cells

**DOI:** 10.1002/iid3.59

**Published:** 2015-05-06

**Authors:** Tomonobu Ito, Chizu Egusa, Tatsuo Maeda, Takafumi Numata, Nobuhiro Nakano, Chiharu Nishiyama, Ryoji Tsuboi

**Affiliations:** 1 Department of Dermatology, Tokyo Medical University6-7-1 Nishi-Shinjuku, Shinjuku-ku, Tokyo 160-0023, Japan; 2 Atopy (Allergy) Research Center, Juntendo University School of Medicine2-1-1 Hongo, Bunkyo-ku, Tokyo 113-8421, Japan; 3 Department of Biological Science and Technology, Tokyo University of Science6-3-1 Niijuku, Katsushika-ku, Tokyo 125-8585, Japan

**Keywords:** IL-33, mast cells, MHC class II, MHC class II transactivator (CIITA), PU.1

## Abstract

Mast cells (MCs), recognized as tissue-resident cells of hematopoietic origin, are involved in cellular and pathological manifestations of allergic disorders including atopic dermatitis. IL-33, a member of the IL-1 cytokine family, activates Th2-type immune responses, and promotes the degranulation and maturation of MCs. However, it is uncertain whether IL-33 treatment induces mature mast cells to acquire the characteristics of the monocyte-dendritic cell lineage.We investigated the effect of IL-33 on the MHC class II expression and function of murine mast cells. IL-33-treated mature murine bone marrow-derived mast cells (BMMCs) were analyzed by FACS, real-time PCR, chromatin immunoprecipitation (ChIP) assay, and Western blotting. The morphology and degranulation activity of BMMCs and T-cell activation by BMMCs were also examined. BMMCs treated with IL-33 for 10 days induced cell surface expression of the MHC class II protein, whereas the expression of FcεRI and c-kit was not affected by IL-33. The expression of CIITA, driven from pIII and pIV, was up-regulated in IL-33-treated BMMCs. The amount of PU.1 mRNA and protein significantly increased in IL-33-treated BMMCs. The ChIP assay showed PU.1 binding to CIITA pIII, and enhanced histone acetylation due to IL-33 treatment. Syngeneic T cells were activated by co-culture with IL-33-treated BMMCs, although the expression of the co-stimulatory molecules, CD40, CD80, CD86, and PDL-1, was not detected. Mast cells express MHC class II after prolonged exposure to IL-33, probably due to enhanced recruitment of PU.1 to CIITA pIII, resulting in transactivation of CIITA and MHC class II. IL-33 is an important cytokine in allergic disorders. Mast cells have the ability to express MHC class II after prolonged exposure to IL-33 in a murine model. IL-33 holds a key to understanding the etiology of atopic dermatitis.

## Introduction

Mast cells (MCs) are recognized as tissue-resident cells of hematopoietic origin, that is, cells that develop in tissues like the skin and mucosa. MCs participate in innate and acquired immune defense mechanisms through the release of an array of inflammatory mediators following receptor-dependent activation. These mediators, however, are also responsible for initiating the cellular and pathological manifestations of allergic disorders including anaphylaxis, asthma, rhinitis, and atopic dermatitis 1,2. MCs are predominantly activated through antigen/IgE-mediated aggregation of the high-affinity IgE receptor (FcεRI). It is apparent that cytokines and other molecules present in the surrounding milieu can acutely amplify MC signaling and responses to the antigen.

IL-33 is a new member of the IL-1 cytokine family that binds to heterodimeric receptors composed of ST2 and IL-1 receptor accessory protein 3,4. IL-33, which is localized in the nucleus of cells in a steady state, is released upon cell lysis following inflammatory stimulation 3,5. IL-33 activates Th2-type immune responses with the enhanced production of IL-4, IL-5, and IL-13, and promotes the degranulation and maturation of MCs and eosinophils 3,6. It is well known that MCs express ST2 strongly 7 and that IL-33 is involved in the development and maturation of MCs 5,8. In a recent report 9, IL-33 promoted dendritic cell development and was also shown to be involved in the development of both mast and dendritic cells. From these observations, we hypothesized that mature mast cells have the potential to acquire certain characteristics of the monocyte-dendritic cell lineage via IL-33-treatment. In the present study, differentiated murine bone marrow-derived mast cells (BMMCs) were found to express MHC Class II proteins strongly by stimulation with recombinant IL-33 for 10 days. We examined the effect of IL-33 on the expression of monocyte- and mast cell-specific markers, cell morphology, and T-cell activation, and analyzed MHC class II transactivator (CIITA) expression in IL-33-treated mature MCs.

## Materials and Methods

### Mice

BALB/c mice were purchased from The Jackson Laboratory (Bar Harbor, ME). Clone DO11.10, ovalbumin-specific T-cell receptor transgenic mice on a BALB/c background which recognizes the 323–339 peptide fragment of ovalbumin, also purchased from The Jackson Laboratory 10 were donated by Dr. Yoshimoto of Tokyo Medical University. All experiments were performed in accordance with the practices of the institutional science community and the national guidelines for animal experimentation, and approved by The Institutional Animal Care and Use Committee (IACUC) of Tokyo Medical University (S-4).

### Cell culture

BMMCs were developed from bone marrow obtained from the femurs of 6–10-week-old Balb/c mice as described 11. Essentially, the cells were cultured for 4–6 weeks in RPMI1640 medium containing 10% FBS, glutamine (4 mM), sodium pyruvate (1 mM), penicillin (100 U/mL), streptomycin (100 mg/mL), non-essential amino acids (Sigma, St Louis, MO), HEPES (25 mM), 2-mercaptoethanol (50 mM), and mouse recombinant IL-3 (20 ng/mL) (Peprotech, Rocky Hill, NJ). The cells were maintained at 37°C in a humidified incubator gassed with 95% air and 5% CO_2_. The purity of the cultures, as assessed by toluidine blue staining 12 and FcεRI expression and KIT expression, was >99%.

### Cell sensitization

BMMCs were sensitized with mouse anti-DNP-IgE (clone SPE-7 100 ng/mL Sigma–Aldrich) overnight in a cytokine-free medium. After sensitization, the cells were washed three times with HEPES buffer (10 mM HEPES [pH 7.4], 137 mM NaCl, 2.7 mM KCl, 0.4 mM Na_2_HPO_4_ · 7H_2_O, 5.6 mM glucose, 1.8 mM CaCl_2_ · 2H_2_O, 1.3 mM MgSO_4_ · 7H_2_O) containing 0.04% BSA prior to conducting activation experiments.

### Degranulation

Degranulation was monitored by the release of the granule component, β-hexosaminidase (β-hex), into the supernatants as described 13. Sensitized cells were aliquoted into individual wells of a 96-well plate (3 × 10^4^ cells/well, 100 μL final volume) and allowed to equilibrate to 37°C for 10 min. Cells were then stimulated with DNP-HSA (0–10 ng/mL) for 30 min in the wells. The reactions were terminated by centrifugation (1600*g* for 5 min) at 4°C. The supernatant was then assayed for β-hex content. The remaining cells were then lysed by adding 0.1% Triton X (Wako, Osaka, Japan) and aliquots were similarly assayed for β-hex content. Degranulation was calculated as the percentage of total β-hex found in the supernatants following challenge.

### Cytokine release

Sensitized or non-sensitized BMMCs (5 × 10^5^ cells/mL) were stimulated with DNP-HSA (10 ng/mL) for 6 h in an IL3-free medium. The cell-free supernatants were harvested and stored at −80°C prior to conducting the cytokine assays. To measure the cytokine retained in the cellular content, harvested cells were disrupted with double distilled water (DDW), left at −80°C for 1 h, then returned to room temperature. Cell lysates were stored at −80°C. Secreted cytokine/chemokine levels were determined using a Duo-set ELISA system (R&D system, Minneapolis, MN, USA) according to the manufacturer's protocol.

### Western blotting

The cells were lysed with a sample buffer (62.5 mM Tris-HCl [pH 6.8], 10% glycerol, 2% SDS, 0.1 mg/mL bromphenol blue dye, and 10% 2-mercaptoethanol). Lysates were sonicated and then boiled for 5 min. Cell lysates were resolved on 4–12% Nupage Bis–Tris gels (Invitrogen, Carlsbad, CA), then transferred to nitrocellulose membranes with the iBlot™ Dry-blotting System (Invitrogen, Paisley, UK) and probed for immunoreactive proteins using the following protein-specific Abs: LaminB (M-20, Santa Cruz Biotechnology, Santa Cruz, CA) and PU.1 (T-21, Santa Cruz Biotechnology). The immunoreactive proteins were visualized by a Western Breeze Chemiluminescent Immunodetection Kit (Invitrogen), and signals were detected with the ChemiDoc XRS system (Bio-Rad, Hercules, CA).

### Flow cytometric analysis of surface and intracellular expression

Following the blocking of Fcγ receptors with 2.4G2 (BD Pharmingen, Franklin Lakes, NJ, USA), cells were stained with PE-anti FcεRI (eBioscience), APC-anti FcεRI (eBioscience), FITC-anti KIT (BD Pharmingen), PE-anti KIT (BD Pharmingen), FITC-anti MHC clssII (I-Ad) (eBioscience), FITC-anti-CD40 (eBioscience), FITC-anti- CD80 (eBioscience), FITC-anti- CD86 (eBioscience), FITC-anti-PD-L1(eBioscience), APC-anti-CD11c (eBioscience) antibody to examine the expression of FcεRI, KIT, MHC class II, CD40, CD80, CD86, and PDL-1. To measure cytoplasmic protein, the cells were fixed and treated with BD Cytofix/Cytoperm™ Plus Fixation/Permeabilization Kit with BD GolgiPlug (BD Bioscience) according to the manufacturer's protocol. The cells were then incubated for 1h at 4°C. After washing with PBS, cells stained with Ab were analyzed using a FACS Calibur flow cytometer (BD Biosciences) and associated CellQuest software.

### May–Giemsa, toluidine blue, and immunofluorescence staining

Cytospin of 4-week-old BMMCs was prepared, fixed, and stained with May–Giemsa and toluidine blue, as described 11. MHC class II-positive cells were isolated by magnetic cell sorting using an anti-MHC class II micro beads mouse (Miltenyi Biotec, Bergisch Gladbach, Germany) according to the manufacturer's instructions. For confocal laser scanning microscopy, the cells (1 × 10^3^ cells) were fixed in 4% paraformaldehyde phosphate buffer solution (PFA) for 20 min at 4°C. After washing with PBS, the cells were stained with PE-anti FcεRI (eBioscience) Ab, FITC-anti KIT (BD Pharmingen), and APC anti-MHC class II (I-Ad) (eBioscience) for 1 h at 4°C. After washing with PBS, the cells were analyzed using confocal laser microscopy (LSM 5 PASCAL, Carl Zeiss, Oberkochen, Germany) in conjunction with LSM image Brower software (Carl Zeiss, Oberkochen, Germany).

### Gene expression analysis

Total RNA was isolated using High Pure RNA isolation Kit (Roche, Basel, Switzerland). A total of 0.5 μg of RNA was used for reverse transcription with a High Capacity cDNA Reverse Transcription Kit (Applied Biosystems, Foster City CA) reacted with random primer in a 20-μL solution. One μL of the resulting cDNA was used for real-time PCR with the primers and probes prepared by the Universal Probe Library Assay Design Center (Roche): murine CIITA common (primers: F 5′-ctcagccaccttccctca-3′, R 5′-cagtgatgttgttttgggaca-3′), CIITA promoter I (primers: F 5′-cccggaagatttccctga-3′, R 5′-tggtggcacacagactatgg-3′), CIITA promoter III (primers: F 5′-gcatcactctgctctctaaatcat-3′, R 5′-tggtggcacacagactatgg-3′), CIITA promoter IV F (primers: F 5′-ctaggagccacggagctg-3′, R 5′-tggtggcacacagactatgg-3′), and PU.1 F (primers: 5′-gggatctgaccaacctgga-3′, R 5′-aaccaagtcatccgatggag-3′). Gene expression was determined based on a comparison of the CT values for the gene of interest and 18S rRNA with the mean CT values for the control group to determine a fold induction value, as previously described 14,15.

### Cell activation with mast cell—CD4^+^ T-cell co-culture

Spleen cells of the DO11.10 mice were prepared in single-cell suspensions and CD4^+^ T cells (purity >96%) were isolated by magnetic cell sorting using a CD4^+^ T-cell Isolation Kit (Miltenyi Biotec) according to the manufacturer's instructions. Matured (4 weeks) BMMCs (1 × 10^5^ cells) that were activated by 50 ng/mL recombinant IL-33 for 10 days were stimulated with 10 μmol/L ovalbumin_323–339_ peptide (Abgent, San Diego, CA, USA) for 24 h in a medium containing IL-3 (20 ng/mL) and IL-33 (50 ng/mL). The cells were treated with 25 μg/mL mitomycin C (MP Biomedicals, Santa Ana, CA, USA) for 30 min at 37°C. After 30 min, the cells were washed three times with BMMC culture medium. Mitomycin C treated BMMCs (1 × 10^5^ cells) were co-cultured with 1 × 10^5^ CD4^+^ T cells in the IL-3-containing BMMC culture medium in black, flat-bottomed, 96-well culture plates for 48 h. After co-culturing, the levels of CD4^+^ T-cell activation were determined using Cell Proliferation ELISA system (Roche) according to manufacturer's protocol. The co-cultured cells were labeled with 10 μL/well BrdU at 37°C for 2 h. The labeled cells were centrifuged at 300*g* for 10 min, washed twice, then removed from the labeling medium and dried using a hairdryer. After cell fixation, the dried, attached cells were added to 100 μL/well anti-BrdU for 90 min at room temperature. After they were washed, the substrate was added for 3 min, and the chemiluminescence was measured using a luminometer (Mithras LB940, Berthold Technologies, Bad Wildbad, Germany).

### Chromatin immunoprecipitation assay

The chromatin immunoprecipitation assay was performed using a ChIP-IT® express kit (Active motif, Carlsbad, CA) according to the manufacturer's instructions with some modifications. Anti-PU.1 rabbit IgG (T-21, Santa Cruz Biotechnology), anti-acetyl histone H3 rabbit IgG (no.39139; Active motif), anti-acetyl histone H4 rabbit IgG (no. 39925; Active motif), and normal rabbit IgG (2729s, cell signaling) were used. Quantitative PCR of chromosomal DNA was performed using the same method as for mRNA quantification with the following primers and TaqMan probes: for the pIII promoter (−205/106), forward primer pIII-205′ -AGGTGCAGCTTCTGTGGTC-3′), reverse primer pIII-106R (5′-CCTCTAATTTGCCCATGC-3′), and TaqMan probe pIIII-171P (FAM-5′-CAAGAAGGAACTGAAATT-3′-MGB); for the pIV promoter (−189/−59), forward primer pIV-189 (5′-AAGCAAACTTGGGTTGCATGT-3′), reverse primer pIV-59R (5′-CCCCTTTCACTTTCTGTCTACACCTT-3′), and TaqMan probe pIV-163P (FAM-5′-CTTCTGAGAAAGCACGTGG-3′-TAMRA). The ratio of a specific DNA fragment in each immunoprecipitate to that fragment in the DNA before immunoprecipitation (input DNA) was calculated from each cycle threshold value and is represented as a percentage.

### Statistical analysis

Data are presented as the mean ± S.E. A two-tailed Student's *t*-test was used for statistical analysis. When the *P*-value was<0.05, the difference was considered to be significant.

## Results

### BMMCs express MHC class II after prolonged exposure to IL-33

**Figure 1:**
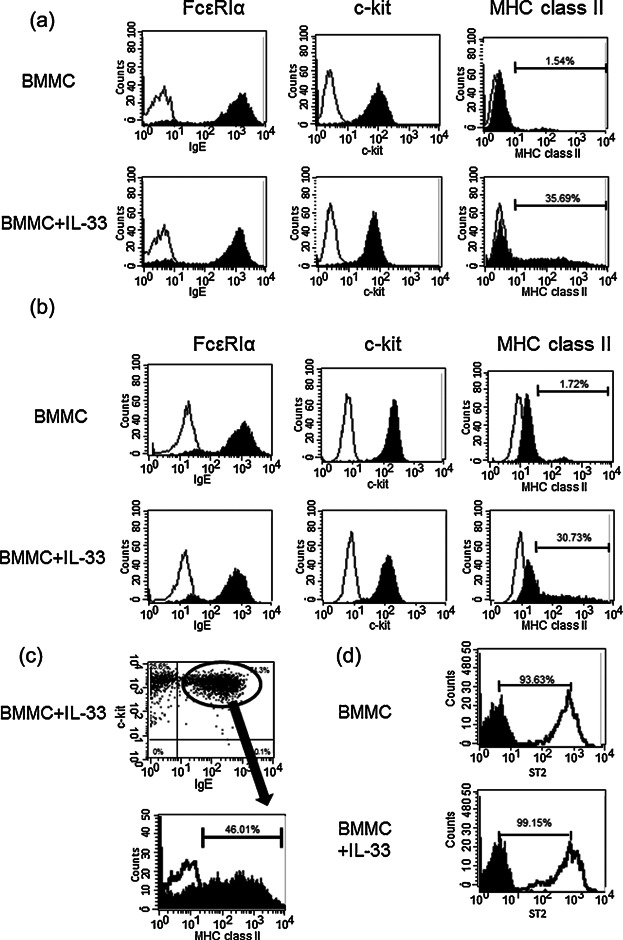
Prolonged exposure of BMMCs to IL-33 induces MHC class II protein. Flow cytometric analysis of the expression level of MHC class II after additional 10 days culture of BMMCs in the presence or absence of IL-33. (a) Cell surface receptors. (b) Intracellular receptors. (c) FcεRI α chain^+^ and c-kit^+^ BMMCs were stained for MHC class II. Shaded histograms: Anti-FcεRI α chain, anti-c-kit, and anti-I-Ad antibodies; unshaded histograms: Hamster IgG2b and rat IgG2b. A representative result of three independent experiments.

**Figure 2:**
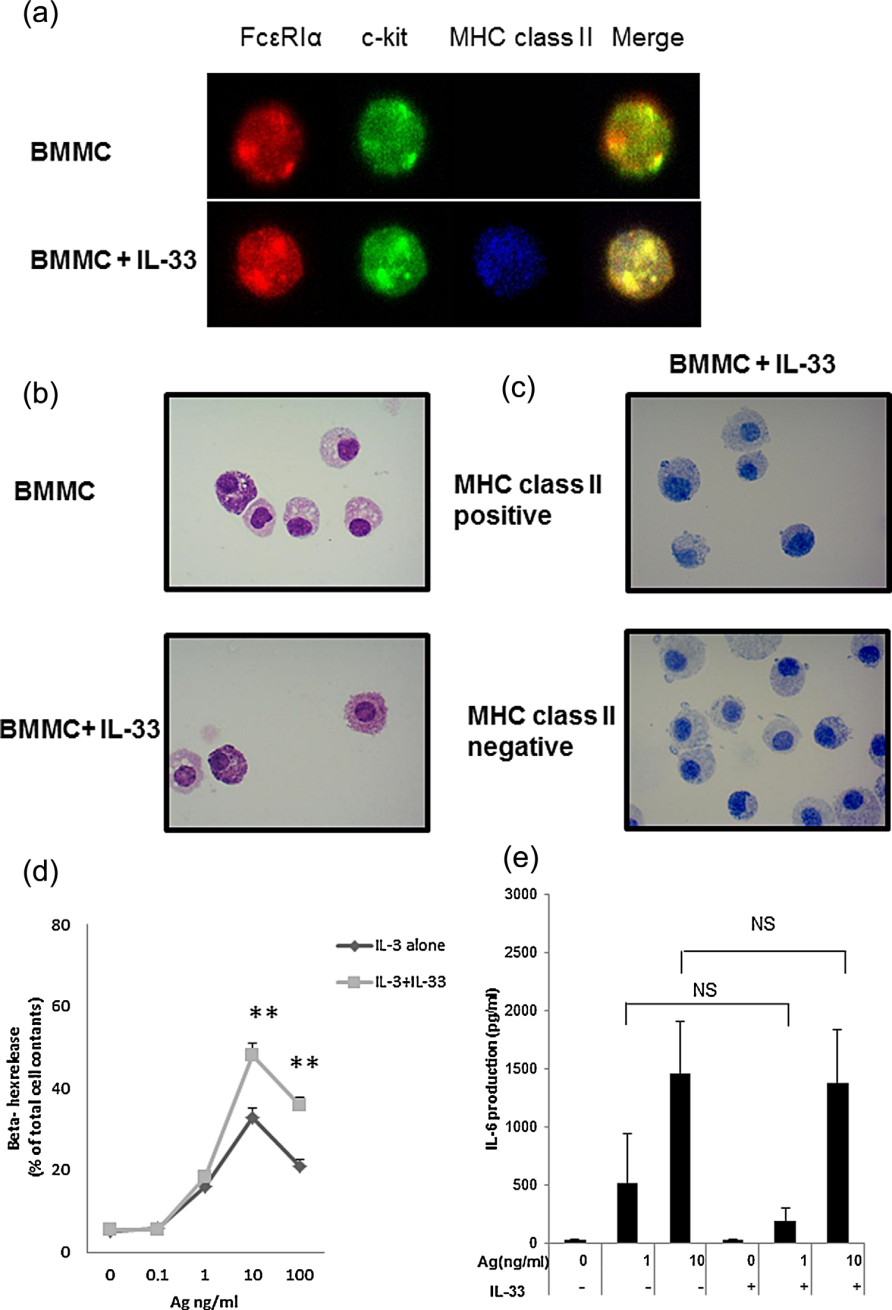
Comparison of MHC class II positive and negative BMMCs. (a) Immunofluorescence staining. Cytospins of BMMCs were fixed in 4% PFA and stained with PE labeled anti FcεRI Ab (red), FITC labeled anti-c-kit Ab (green), and allophycocyanin anti- I-Ad Ab (blue). (b) Cytospins of BMMCs were visualized by May–Giemsa stain. (c) BMMCs exposed to IL-33 were isolated by magnetic cell sorting using the anti-I-Ad microbeads. Collected MHC class II-positive or negative cells were visualized by toluidine blue stain. The images were representative of three experiments. (d) Degranulation level of IL-33-exposed mature BMMCs. BMMCs cultured for 4 weeks in an IL-3-containing medium was stimulated with IL-33 (50 ng/mL) for additional 10 days. The cells were sensitized with IgE overnight in starved conditions. The cells were challenged with the indicated concentrations of Ag (DNP-HSA) for 30 min, and degranulation (β-hex) was assessed. (e) IL-6 production from the IL-33-exposed BMMCs. The IgE-sensitized cells in the cytokine-free medium were then challenged for 6 h with Ag (DNP-HSA) and the amount of IL-6 released into the medium was determined. Data represent mean and SEM (*n* = 3), and differences between the BMMCs and IL-33-treated BMMCs are indicated. **P < 0.01, *t*-test.

The BMMCs were cultured in media containing IL-3 (20 ng/mL) for up to 4 weeks. Over 95% of the population expressed FcεRI α chain and were c-kit positive (data not shown). The BMMCs cultured for 4 weeks with IL-3-containing media were further stimulated with IL-33 (50 ng/mL) for an additional 10 days. The medium was replaced with a fresh medium containing IL-3 and IL-33 every other day. FACS analysis showed that cell surface and intracellular expression of MHC class II was increased by the addition of IL-33, whereas the expression of FcεRI α–chain and c-kit was unaffected (Fig. 1a,b). FcεRI α–chain and c-kit positive pure MCs expressed MHC class II on the cell surface after prolonged IL-33 treatment (Fig. 1c). ST2 receptor which was IL-33 receptor was unaffected (Fig. 1d). Examination by confocal laser scanning microscopy showed that IL-33-treated BMMCs expressed MHC class II, FcεRI α–chain, and c-kit receptors on the cell membrane (Fig. 2a). Morphological difference was not observed between IL-33-treated and non-treated BMMCs. These data suggest that BMMCs persistently exposed to IL-33 have the ability to express MHC class II, and display the morphology of mature BMMCs.

### IL-33-treatment upregulates IgE-mediated degranulation activity of BMMCs

Mast cells exposed briefly to IL-33 reportedly showed enhance Ag/IgE-mediated degranulation and cytokine production 16–19. In contrast, a recent study reported that prolonged exposure to IL-33 during development of MCs down-regulated the degranulation level in human CD34^+^ peripheral blood-derived MCs and BMMCs 20. In the present study, however, the Ag/IgE-mediated degranulation level was up-regulated by IL-33 treatment (Fig. 2d), while the Ag/IgE-mediated IL-6 production level was unaffected (Fig. 2e).

### Time course of MHC class II expression levels in mature BMMCs after exposure to IL-33

**Figure 3:**
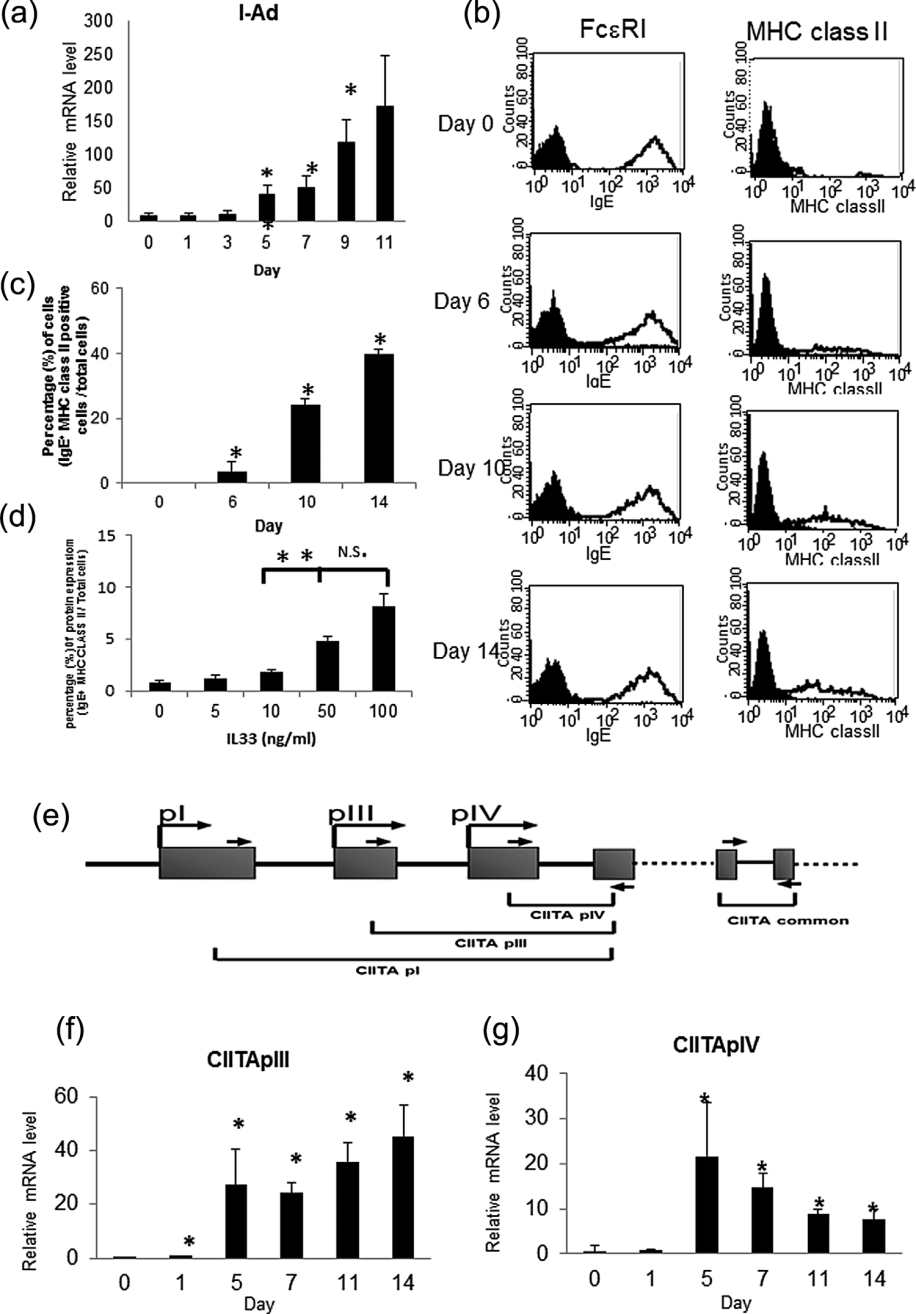
MHC class II expression occurs in mature BMMCs after 5 days of exposure to IL-33. BMMCs cultured in IL-3 alone for 4 weeks were incubated with IL-33 for the periods indicated. (a) I-Ad mRNA expression by real-time PCR. (b) Flow cytometric analysis of the surface expression levels of FCεRI α chain and MHC class II using PE labeled anti-FCεRI α chain and FITC-labeled I-Ad antibodies. Unshaded histograms: Anti- FCεRI α chain and anti-I-Ad antibodies; shaded histograms: Hamster IgG2b and rat IgG2b. A representative result of three independent experiments is shown. (c) The percentage of MHC class II expressed cells under IL-33 stimulation for the periods indicated. The average of MHC class II positive cells is indicated by dot blots. (d) The percentage of MHC class II expressed cells under IL-33 stimulation(0–100 ng/mL) with 7 days for dose-dependent induction. (e) Structure of mouse CIITA gene. (f,g) Relative expressions of CIITA mRNA measured by real-time PCR as a ratio to that of IL-33 stimulated BMMCs for types III and IV. A representative result of three independent experiments is shown (a,b). Data represent the mean and SEM [(c) *n* = 4; (d,f,g) *n* = 3], and differences between BMMCs and IL-33-treated BMMCs are indicated. *P < 0.05, **P < 0.01, *t*-test.

We analyzed the kinetics of IL-33-mediated MHC class II expression by monitoring the I-Ad mRNA levels and MHC class II protein levels in mature BMMCs. I-Ad mRNA expressions significantly increased from days 5 to 11(Fig. 3a). When cell surface MHC class II expression was monitored in cells cultured for 14 days in the presence of IL-33, MHC class II positive cells were detected on day 6 (Fig. 3b). The percentage of MHC class II positive BMMCs increased from 24% on day 10 up to 40% on day 14 (Fig. 3c). The analysis of dose-dependent induction of MHC class II expression was depending on IL-33 concentration on day 7(Fig. 3d). The CIITA gene regulates the MHC class II gene expression in dendritic cells and B cells. The mouse CIITA gene possesses three promoters, pI, pIII, and pIV, which are trans-activated in a cell-type-specific manner (Fig. 3e). CIITA type III mRNA was slightly detected on day 1 and the amount of type III and type IV mRNAs was markedly elevated from days 5 to 14(Fig. 3f,g). These data indicate that approximately 5 days are required for IL-33-mediated expression of MHC class II following CIITA transcription.

### IL-33-induced expression of MHC class II is partly reversible

**Figure 4:**
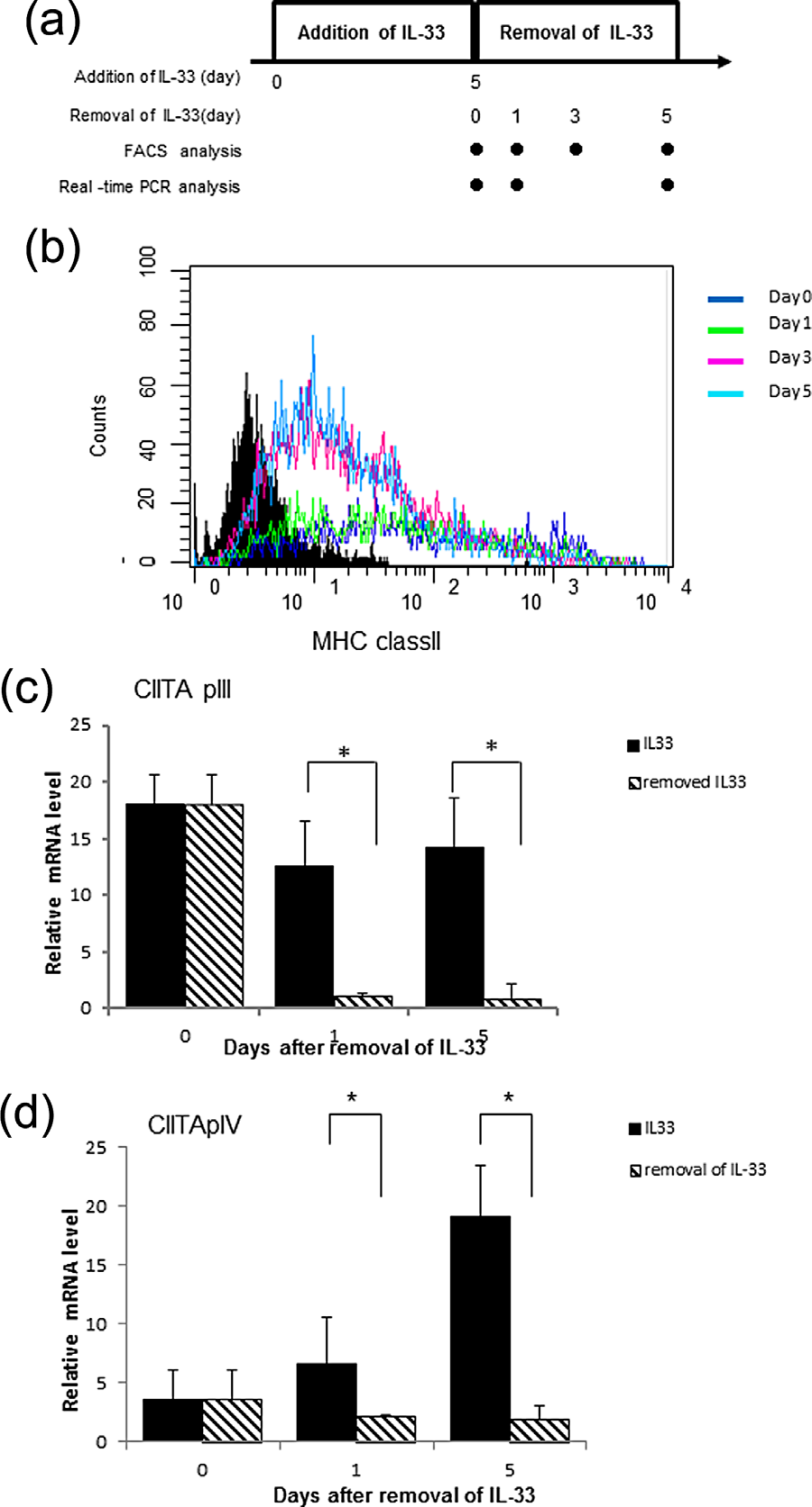
IL-33-induced expression of MHC class II is partly reversible. (a) The schedule of experiments. IL-33 was added to culture medium for 5 days, and later removed. BMMCs were analyzed by FACS and real-time PCR on the day indicated. (b) Flow cytometric analysis of the surface expression level of MHC class II using FITC labeled I-Ad antibody. Shaded histograms are the isotope control. Immediately after removal of IL-33 on day 0 (blue line), day 1; absence of IL-33 for 1 day (green line), day 3; absence of IL-33 for 3 days (pink line) and day 5; absence of IL-33 for 5 days (sky blue line). A representative result of three independent experiments is shown. (c,d) Kinetics of reversible of I-Ad mRNA measured by real-time PCR. mRNA expression of CIITA types III and IV was measured in the absence of IL-33 for 1 and 5 days. A representative result of three independent experiments is shown. Data represent the mean and SEM [(c,d) *n* = 3)], and differences between BMMCs and IL-33-treated BMMCs are indicated. *P < 0.05, *t*-test.

We next examined whether the MHC class protein and CIITA gene expression could be reversed by removing the IL-33, since the effect of IL-33 on enhanced degranulation of human MCs was reduced after removal of IL-33 in our previous report 20. The experimental protocol is briefly shown in Fig. 4a; IL-33 was removed from the culture medium after incubation for 5 days. As shown in Fig. 4b the IL-33-dependent up-regulation of MHC class II decreased in the absence of IL-33 on day 3. Messenger RNA levels of CIITA types III and IV dramatically decreased on day 1 after starvation of IL-33 (Fig. 4c,d). These data demonstrate that enhanced expression of CIITA mRNA is rapidly reduced in the absence of IL-33, causing subsequent down-regulation of MHC class II protein.

### Functional profiles of IL-33 treated BMMCs

**Figure 5:**
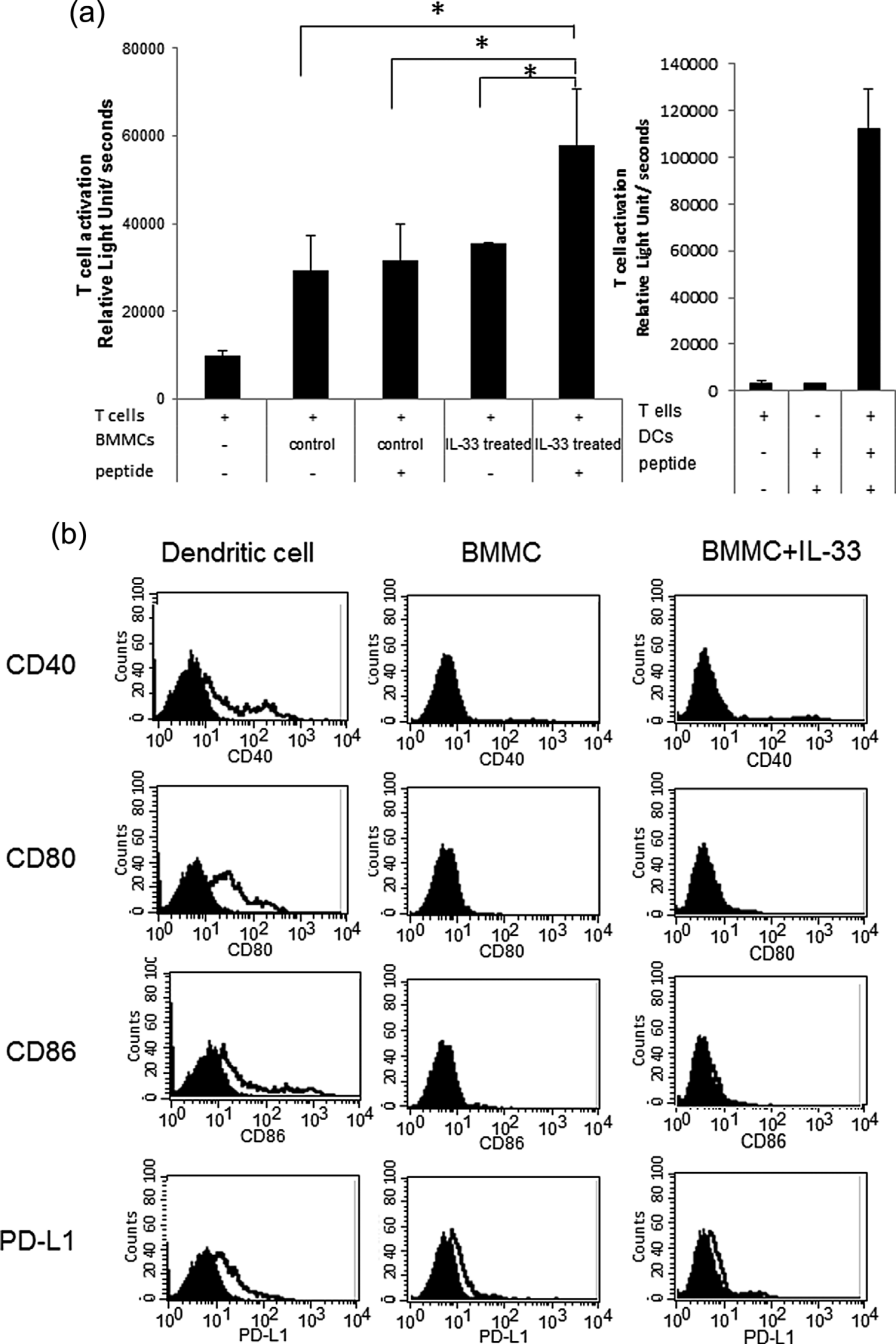
Functional profiles of IL-33- treated BMMCs. (a) CD4^+^ T-cell activation mediated by IL-33-stimulated BMMCs. The CD4^+^ T cells from DO11.10 mice were co-cultured with BMMCs or IL-33-stimulated BMMCs in the presence or absence of 10 μmol/L ovalbumin_323–339_ peptide. Positive controls are used bone marrows dendritic cells which are cultured with IL-4 and GM-CSF for 10 days. Data are indicated as mean + SEM. of triplicate samples. *P < 0.05 versus corresponding values for T cells in the presence of ovalbumin- and IL-33- treated BMMCs (relative light unit/seconds). (b) Flow cytometric analysis of the surface expression level of CD40, CD80, CD86, and PD-L1 by using FITC labeled-antibodies. Unshaded histograms: Anti-CD40, anti-CD80, anti-CD86, and anti-PD-L1 antibodies; shaded histograms: Hamster IgG2b and rat IgG2b. Positive control is CD11c^+^ sorted bone marrow derived dendritic cells which were cultured with recombinant IL-4 (10 ng/mL) and GM-CSF (10 ng/mL) for 7 days. The numbers on each histogram indicate the mean fluorescence intensity and cell counts. A representative result of three independent experiments is shown.

Prolonged exposure of BMMCs to IL-33 induced marked expression of MHC class II, which is a hallmark of APCs (Fig. 1a). Therefore, we set out to determine whether IL-33-treated BMMCs could exhibit stronger antigen presenting activity than non-treated BMMCs. The activation of CD4^+^ T cells was significantly enhanced by IL-33 treatment comparted with non-treated BMMCs enhanced T-cell activity (Fig. 5a). We subsequently examined the expression of the co-stimulatory molecules, CD40, CD80, CD86, and PD-L1 on IL-33-treated BMMCs, and found that exposure of BMMCs to IL-33 failed to induce expression of CD40, CD80, CD86, and PD-L1, which were detected on bone marrow derived dendritic cells (Fig. 5b). These data demonstrate that IL-33-treated BMMCs can activate T cells, but do not express co-stimulatory molecules of APCs.

### PU.1 is recruited to the CIITA pIII and IV promoters after prolonged exposure of BMMCs to IL-33

**Figure 6:**
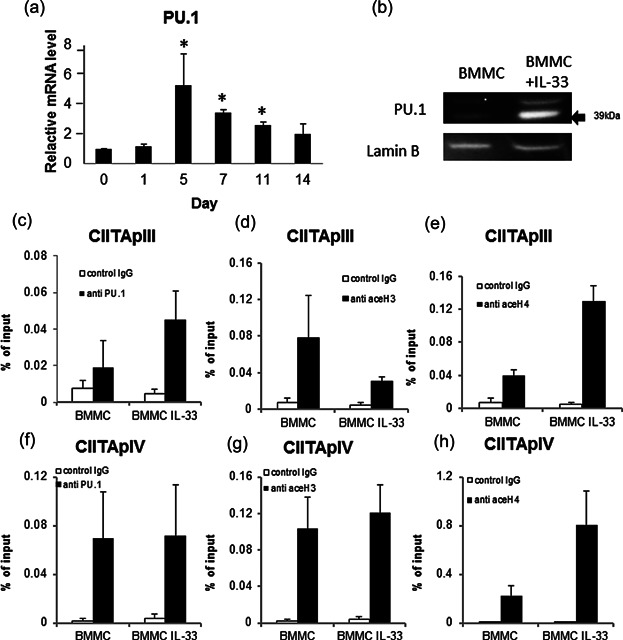
PU.1 was recruited to the CIITA pIII and pIV promoters after prolonged exposure of BMMCs to IL-33. (a) PU.1 mRNA expressions measured by real-time PCR as a ratio to that of untreated BMMCs on day 0. Data represent the mean and SEM (*n* = 3), *P < 0.05, *t*-test. (b) BMMCs and IL-33-treated BMMCs were lysed, and PU.1 expression was analyzed by immunoblotting. ChIP assays were used for quantitative analysis of PU.1 binding to the CIITA pIII promoter (c), and the acetylation status of histones H3 (d) and H4 (e) in the pIII promoter. Analysis of PU.1 binding to the CIITA pIV promoter(f), and the acetylation status of histones H3 (g) and H4 (h) in the pIV promoter. Solid bar: Specific antibody; open bar: Isotype control (rabbit IgG). Three independent experiments with triplicate samples.

The role of PU.1 in the transactivation of the CIITA pIII promoter in B cells is well documented 21,22. We previously found that Notch signaling induced MHC class II expression on MCs via enhanced recruitment of PU.1 on the CIITA pIII promoter 23,24. Therefore, we examined the effect of IL-33 on PU.1 expression in BMMCs. Messenger RNA levels of PU.1 were increased from days 5 to 14 after stimulation with IL-33 (Fig. 6a). Western blotting showed that the PU.1 protein level in whole MCs was up-regulated by IL-33 stimulation (Fig. 6b). ChIP assay showed that the amount of PU.1 binding to the pIII promoter region increased through IL-33 treatment (Fig. 6c). Furthermore, analysis of the histone acetylation status of the pIII promoter region showed that IL-33 treatment increased the acetylation of H4, but not of H3 (Fig. 6d,e). Meanwhile, the amounts of PU.1 binding to the pIV promoter (Fig. 6f) remained unchanged, and the degree of H3 and H4 acetylation around the pIV promoter region (Fig. 6g,h) increased slightly in IL-33-stimulated BMMCs. These results demonstrate that PU.1 expression is up-regulated by IL-33 treatment, resulting in enhanced recruitment of PU.1 to the CIITA pIII, but not to the pIV, promoter. MHC class II mRNA transcription is reflected by H4 acetylation of the pIII and pIV promoter.

### NF-κB and AP-1 have the ability of regulated to MHC class expression by IL-33

**Figure 7:**
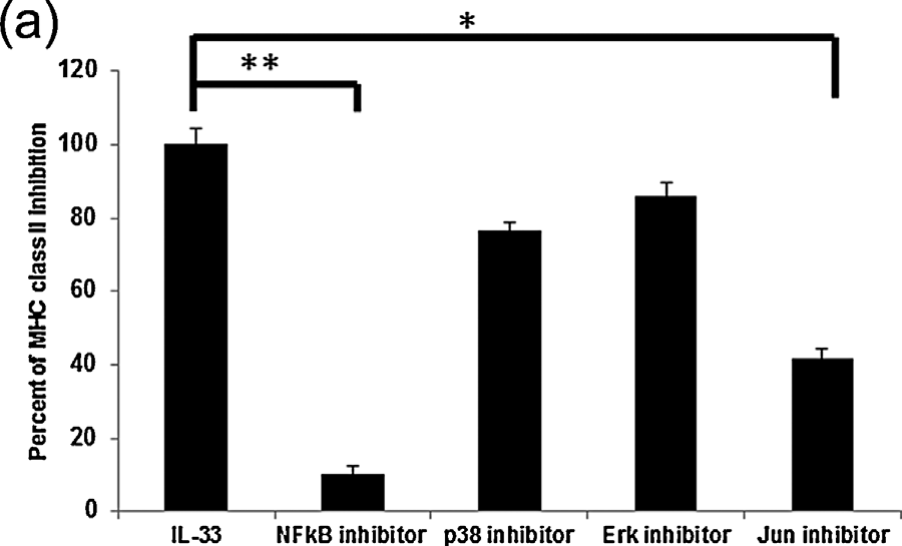
NF-κB and AP-1 have the ability of regulated to MHC class expression by IL-33. JNK inhibitor: SP600125 (Sigma–Aldrich) 10 μM or NF-κB sigma inhibitor: IKK Inhibitor VII (Calbiochem®, Darmstadt, Germany) 2 or 10 μM Erk inhibitor: U0126 (cell signaling) or 10 μM p38 inhibitor: SB203580 (Cayman Chemical Company, Ann Arbor, MI, USA) were stimulated 48hour during IL-33 (50 ng/mL) stimulation at 5 days. The data were percentage of inhibition of MHC class II expression. BMMC+IL-33 stimulated cells were as maxim control (100%). Data represent the mean and SEM and differences between IL-33-treated and IL-33^+^ inhibitor reated BMMCs. BMMCs are indicated. *P < 0.05, * *P < 0.01, *t*-test.

IL-33 receptor leads to the activation of two key transcription factors of the AP-1 pathway through the JNK and NF-κB pathway through IKK from this complex. BMMCs were inhibited by NF-κB, JNK, p38, and Erk under IL-33 stimulation. MHC class II expression was decreased to inhibition of NF-κB, JNK (Fig. 7).

## Discussion

IL-33 activates the Th2-type immune response and pro-inflammatory cytokines, which are elevated in serum and tissue in atopic dermatitis 24,25, chronic asthma 5,26, and allergic rhinitis 27–29. Repeated cutaneous injections of IL-33 increased the number of infiltrated mast cells in BALB/c and C57BL/6 mice 30,31. In addition, short-term IL-33 treatment of BMMCs induced degranulation 18,19,32,33, whereas long-term IL-33 treatment had the opposite effect 20. The present study is the first to demonstrate that long term IL-33 treatment induces expression of MHC class II proteins in mature BMMCs.

Previous studies reported that spleen-derived mast cells expressed MHC class II protein after stimulation by LPS and IFN-γ 34. Immature BMMCs reportedly expressed MHC class II protein and acquired antigen-presenting activity, which diminished after maturation 35. In our experiments, mature BMMCs cultured for 4 weeks expressed MHC class II protein only very slightly (Fig. 1a,b). On the other hand, mature BMMCs cultured for 4 weeks in the presence of IL-33 expressed MHC Class II protein strongly (Fig. 1). We speculate that mature BMMCs are able to express monocyte-specific proteins under specific cell culture conditions. We excluded from the culture medium factors known to induce properties of dendritic cells, or monocytes, such as GM-CSF and IL-4 36, and, with the exception of the presence of IL-33, always employed the same culture conditions as the control.

As shown in Fig. 2d, IL-33-treated mature BMMCs significantly increased FcεRI-mediated MC degranulation. However, no difference was observed in Ag-IgE-mediated IL-6 production between IL-33-treated and IL-33-untreated BMMCs (Fig. 2e). Previous reports have demonstrated involvement of the PLCγ signaling pathway in FcεRI-mediated degranulation of MCs 37. Signaling pathways for cytokine production, such as MAPKs, were weakened in IL-33-treated MCs 20. In our experiment, non-treated BMMCs showed slightly enhanced T-cell activity ovalbumin_323–329_ peptide-independently (data not shown), while IL-33-treated BMMCs showed significantly activation of naïve CD4+ T cells from DO11.10 mice in the presence of ovalbumin_323–339_ peptide (Fig. 5a). The mechanism of CD4+ T-cell activation by IL-33-treated BMMCs may differ from that mediated by DCs because IL-33-treated BMMCs did not express OX40L, CD80, CD86, or PD-L1 (Fig. 5b).

MHC class II expression is an important hallmark of professional antigen-presenting dendritic cells and thymic epithelial cells. In other non-hematopoietic cells, including fibroblasts, astrocytes, endothelial cells, epithelial cells, and keratinocytes, treatment by IFN-γ induces MHC class II gene expression on the cells. The cell type- and cytokine-specific expression of MHC class II gene is regulated by a cofactor termed class II transactivator (CIITA) 38. Human and murine CIITA genes possess 4 (pI, pII, pIII, and pIV) and 3 (pI, pIII, and pIV) cell type-specific promoters, respectively 39. Previous studies of the murine CIITA gene indicated that pI, pIII, and pIV are mainly used in DCs, B cells, and IFN-γ-stimulated cells, respectively 39–41. The present study demonstrated that IL-33-treated BMMCs up-regulated the mRNA expression of CIITA pIII and pIV (Fig. 3f,g) while other studies have reported that IFN-γ induced IL-33 expression in human keratinocytes 42,43.

Transcription factors such as E47, IRF-4, and PU.1 are known to co-regulate CIITA pIII expression in B lymphocytes 21,22,44. PU.1 plays a role in CIITA-pIII activation by binding to a distal element that modifies the chromatin conformation necessary for CIITA expression 22. In our previous studies, we reported that the expression of certain monocyte/DC-specific genes, including MHC class II, was induced in mast cells with highly expressed PU.1 45,46. In the present study, we demonstrated that prolonged exposure of BMMCs to IL-33 increased mRNA and protein levels of PU.1 (Fig. 6a,b). PU.1 constitutively binds to the CIITA pIII promoter. In view of the fact that PU.1 is present in several forms with apparently varying molecular masses (from 38.5 to 46.5 kDa) 47, it is likely that the proteins recognized by anti-PU.1 Ab were PU.1 isoforms. In addition, IL-33 was found to induce up-regulation of CIITA transcription and translation by H4 acetylation of the pIII promoter region (Fig. 6e), and subsequently to up-regulate MHC class II mRNA transcription. The IL-33 receptor leads to the activation of two key transcription factors, namely the AP-1 pathway mediated by JNK and the NF-κB signaling pathway mediated by IKK. When BMMCs were inhibited by NF-κB and JNK under IL-33 stimulation, MHC class II expression decreased (Fig. 7). However, we could not determine the cause of decrease MHC class II expression by IL-33 between MAPK and PU.1.

The primary focus of our results rests on the question of whether the induction of CIITA and MHC class II expressions occurred due to the direct effect of IL-33 or its indirect effect via mediation by other secreted secondary cytokines. As shown in Fig. 4, following removal of IL-33 after 5 days' incubation, MHC class II protein, CIITA pIII, and pIV expressions diminished. Replacing the culture medium every other day with a fresh medium containing the indicated doses of IL-33 induced MHC class II protein expression, suggesting that IL-33 directly affected the BMMCs (Fig. 1). We were unable to detect IFN-γ mRNA expression in the IL-33-treated BMMCs (data not shown). Treatment of BMMCs with TNF-α did not induce MHC class II expression. Furthermore, the neutralizing antibody against TNF-α in IL-33-treated BMMCs diminished MHC class II expression (data not shown). Therefore, as far as we could ascertain, the effect of IL-33 on the expression of MHC class II protein in BMMCs is an IL-33-dependent direct effect. However, we are not certain why prolonged IL-33 exposure is crucial to this process. IFN-γ has been known to induce MHC class II protein in spleen-derived mast cells 35. However, in our study prolonged treatment of BMMCs with IL-33 did not result in IFN-γ secretion. We suggest that the CIITA-activated pathway in mast cells may not only be IFN-γ dependent but also IL-33 dependent. Further studies on the interaction of mast cells with IL-33 will contribute to elucidating the etiology of, and to finding new therapeutic agents for, allergic disorders, especially atopic dermatitis.
